# Multiple Myeloma-Like Spinal MRI Findings in Skeletal Fluorosis: An Unusual Presentation of Fluoride Toxicity in Human

**DOI:** 10.3389/fonc.2016.00245

**Published:** 2016-11-21

**Authors:** Javed Ahsan Quadri, Mohd Meraj Alam, Saba Sarwar, Ashraf Ghanai, A. Shariff, Taposh K. Das

**Affiliations:** ^1^Anatomy, All India Institute of Medical Sciences (AIIMS), New Delhi, India; ^2^Surgery, Guwahati Medical College and Hospital, Guwahati, India; ^3^Endocrinology, Metabolism and Diabetes, All India Institute of Medical Sciences (AIIMS), New Delhi, India

**Keywords:** dual-energy X-ray absorptiometry, fluorotic myelopathy, MRI, M-protein electrophoresis, neoplastic bone marrow infiltration, spinal fluorosis

## Abstract

Endemic fluorosis is a worldwide environmental problem due to excessive fluoride, commonly due to increased drinking water fluoride levels but sometimes due to other sources such as food with high fluoride content. In India, 21 of the 35 states are known to have health problems associated with fluoride toxicity. The present report is a case of a 50-year-old female who was seen with progressive spinal complications and a MRI of the spine suggestive of multiple myeloma. The MRI of the lumbosacral spine showed a diffuse and heterogeneous marrow signal of the lower dorsal and lumbosacral vertebrae. The MRI was also suggestive of coarse trabeculation and appeared predominantly hypointense on the T1W image and had mixed signal intensity on the T2W image. These findings were suggestive of neoplastic bone marrow infiltration and the presence of a proliferative disorder, with multiple myeloma being the most likely. During the patient workup, it was found that other family members were also having similar complications and, after investigation of these family members, it was found that they are suffering from systemic fluorosis. The patient was then evaluated for skeletal fluorosis, and this condition was found to be present. Multiple myeloma was ruled out by the finding of a negative serum protein electrophoresis. The spinal complications appeared to be mainly due to the compression of the spinal cord and nerve roots by protruding osteophytes, thickening of the posterior longitudinal ligament, and thickening of the ligamentum flavum resulting in a compressive myeloradiculopathy and compressive myelopathy. The finding of multiple myeloma-like findings on the spinal MRI in association with skeletal fluorosis was considered to be a very rare event. This case report underlines the need to consider the presence of spinal skeletal fluorosis when evaluating spinal complications with unusual pseudo-multiple myeloma-like changes on the spinal MRI.

## Introduction

Endemic skeletal fluorosis is widely prevalent in India, China, and in many other countries around the world (1). The primary findings of fluorosis are mottling of teeth, osteosclerosis, soft tissue calcification, and marginal bony overgrowth (2). Symptoms range from mild motor and sensory loss to spastic paraplegia and spastic quadriplegia with bladder and bowel incontinence. Association of fluoride toxicity with dysfunction of bone, and endocrine, gastrointestinal, and reproductive systems have been previously reported (3–5). Effects of fluoride on corticosteroids metabolism and its role in bone mineralization have also been reported by Das et al. In this case report, we are reporting very rare multiple myeloma-like MRI findings in a skeletal fluorosis patient.

## Case Report and Description

We are presenting a case report of a 50-year-old female from a village of Agra, in the state of Uttar Pradesh, an endemic fluorosis region in India. Patients belong to lower socioeconomic strata, and the source of income is agriculture. Patient was referred from district hospital to the supper specialty cancer hospital for the treatment of multiple myeloma, on the basis of radiological (MRI spine) findings. Patients represented with hemogram values as: hemoglobin 9.0 g/dl (12.0–15.0), RBC 3.61 × 10^6^/μl (4.0–4.9), HCT-28.2% (36–44), MCV-78.1fL (80–100), MCH-24.9 g/dl (26–34), RDW-18.7% (<14.5), and platelets count was normal (268 × 10^3^/μl), while ESR 40 mm (normal value for women over 50 years old: <30 mm/h) was significantly raised after 1 h, alkaline phosphatase was increased to 733 IU/l (33–131 IU/l). Lymphoma was ruled out, because lymphoma patients usually present with symptoms such as lymphadenopathy, night sweats, weight loss, dyspnea, itching, etc. All these symptoms were absent in the patient accept mild to moderate weight loss. The chances of renal osteodystrophy were negligible because osteodystrophy is a bone disease that occurs when patient’s kidneys fail to maintain proper levels of calcium and phosphorus in the blood. The blood calcium level in the subject was 9.1 mg/dl (normal range 8.1–10.4 mg/dl), renal osteodystrophy was ruled out. Because the serum level of PTH was in normal 49.06 pg/ml (normal range 15–65 pg/ml), hyperparathyroidism was least likely.

**Figure 1:**
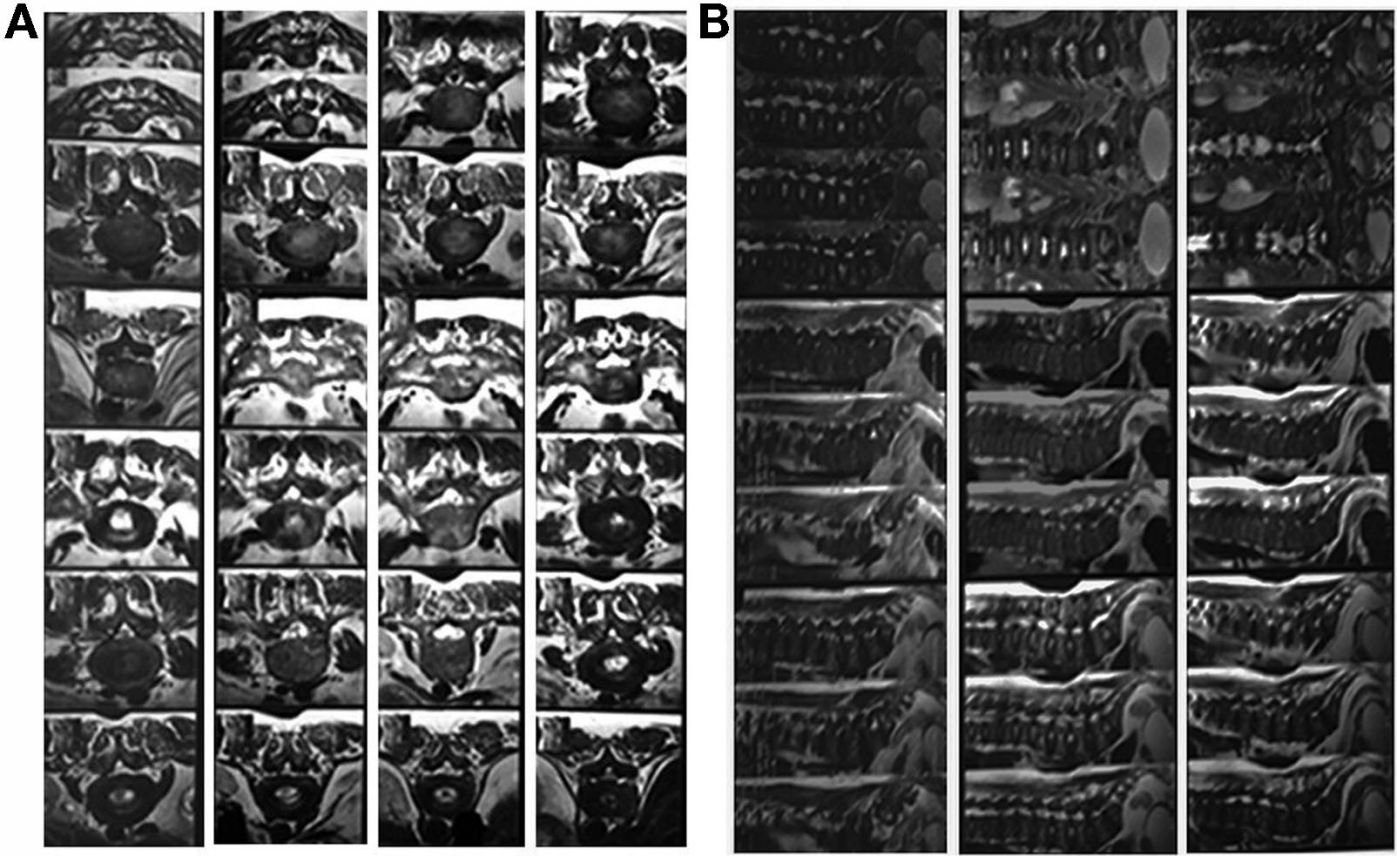
**(A,B)** MRI findings showing multiple myeloma-like changes.

The patient was advised for MRI spine to diagnose the cause of severe spinal pain and stiffness. MRI of the lumbosacral spine showed a diffusely and heterogeneous marrow signal of visualized lower dorsal and lumbosacral vertebrae, their posterior elements as well as pelvic bones. MRI was also suggestive of coarse trabeculation and appears predominantly hypointense on T1W and mixed signal intensity on T2W image. These findings were suggestive of neoplastic bone marrow infiltrative and proliferative disorders and are more in favor of multiple myeloma rather than metastasis and other disease (Figures 1A,B). The MRI findings such as appearance of normal ligamentum flavum with no hypertrophy and normal appearance of pre- and paraspinal soft tissue were not suggestive of skeletal fluorosis also.

## The Sequence of MRI Findings of Lumbosacral Spine

Lumbosacral spine was examined in the sagittal, coronal, and axial planes as per the standard protocol. Both T1- and T2-weighted images were obtained. Additionally, fat sat sagittal T2 images were also obtained. Diffusely altered and heterogeneous marrow signals of visualized lower dorsal and lumbosacral vertebrae and their posterior elements as well as pelvic bones were observed. These show coarse trabeculation and appear predominantly hypointense on T1W and mixed signal intensity on T2W images. A remarkably reduced, L2–5 vertebral body height, mid-body wedging, and biconcave disk margins were noted. Concentric disk bulge (wide-based posterior central) protrusion at L4–5 causing thecal sac compression, bilateral neural foraminal narrowing, and compression of bilateral traversing L5, exiting L4, and cauda equina nerve roots. The MRI findings were also suggestive of concentric disk bulge at L3–4 causing thecal sac indentation, bilateral neural foraminal narrowing and bilateral exiting of L3 nerve roots. The disk bulge at L1–2 and L2–3 causing thecal sac indentation and bilateral neural foraminal narrowing observed. Central canal stenosis was seen from D11 to S1. Pre-thecal epidural fat was well maintained without any significant abnormality. Thecal sac shows normal appearance at rest of the levels. The conus medullaris and cauda equina also appeared normal. There is no focal area of cord expansion or cord edema seen. No intraspinal mass is seen. Neural foramina and exiting nerve roots appear normal at rest of the levels. Articular facet and facet joints are normal. No ligamentum flavum hypertrophy was identified. Pre- and paraspinal soft tissue appeared normal.

**Table:** 

Saggital diameter of bony spinal canal	Size in mm
D11–12	02
D12	10
D12–L1	05
L1	10
L1–L2	04
L2	09
L2–3	04
L3	07
L3–4	04
L4	06
L4–5	04
L5	07
L5–S1	04

## Impression of MRI

Diffusely altered and heterogeneous marrow signals of visualized lower dorsal and lumbosacral vertebrae, their posterior elements as well as pelvic bones were observed. This show coarse trabeculation and appears predominantly hypointense on T1W and mixed signal intensity on T2W images. These are suggestive of neoplastic marrow infiltrative/proliferative disorders and are more in favor of multiple myeloma rather than metastasis. Clinicopathological and biochemical correlations were advised.

## Diagnosis and Confirmation of Skeletal Fluorosis

**Figure 2:**
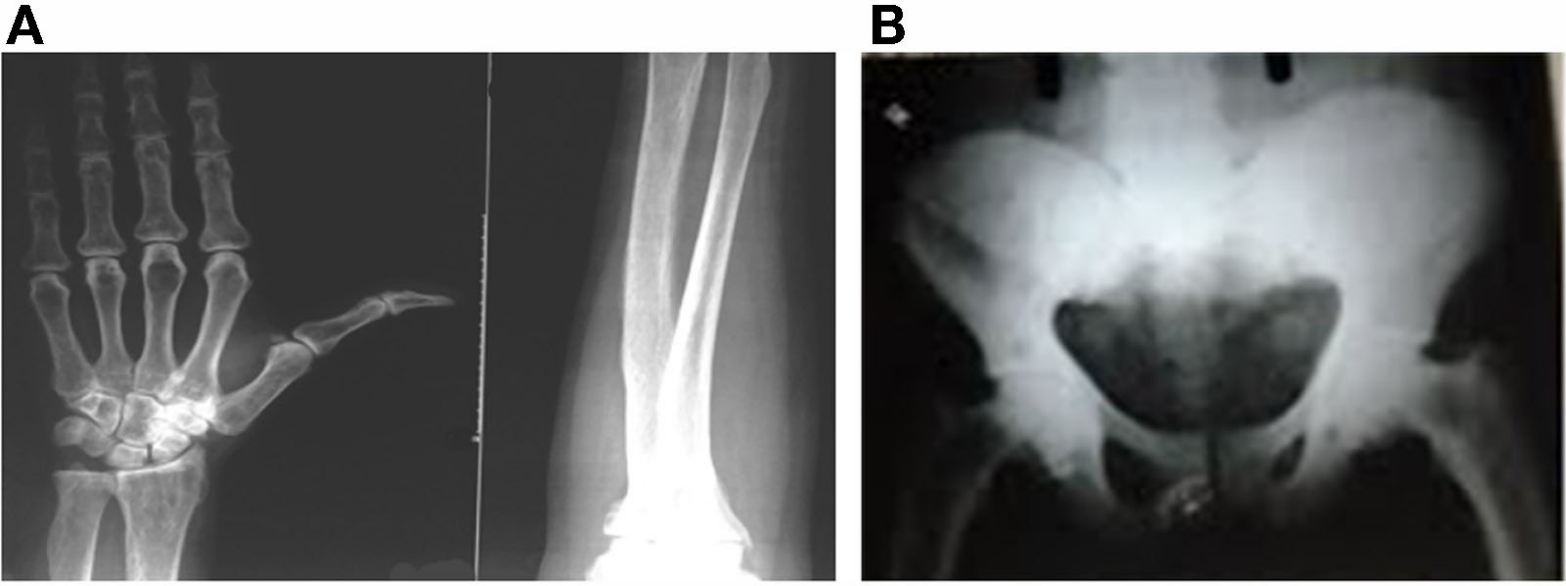
**X-ray, (A) forearm showing interosseous membrane calcification, (B) X-ray hip showing ectopic calcification**.

**Figure 3:**
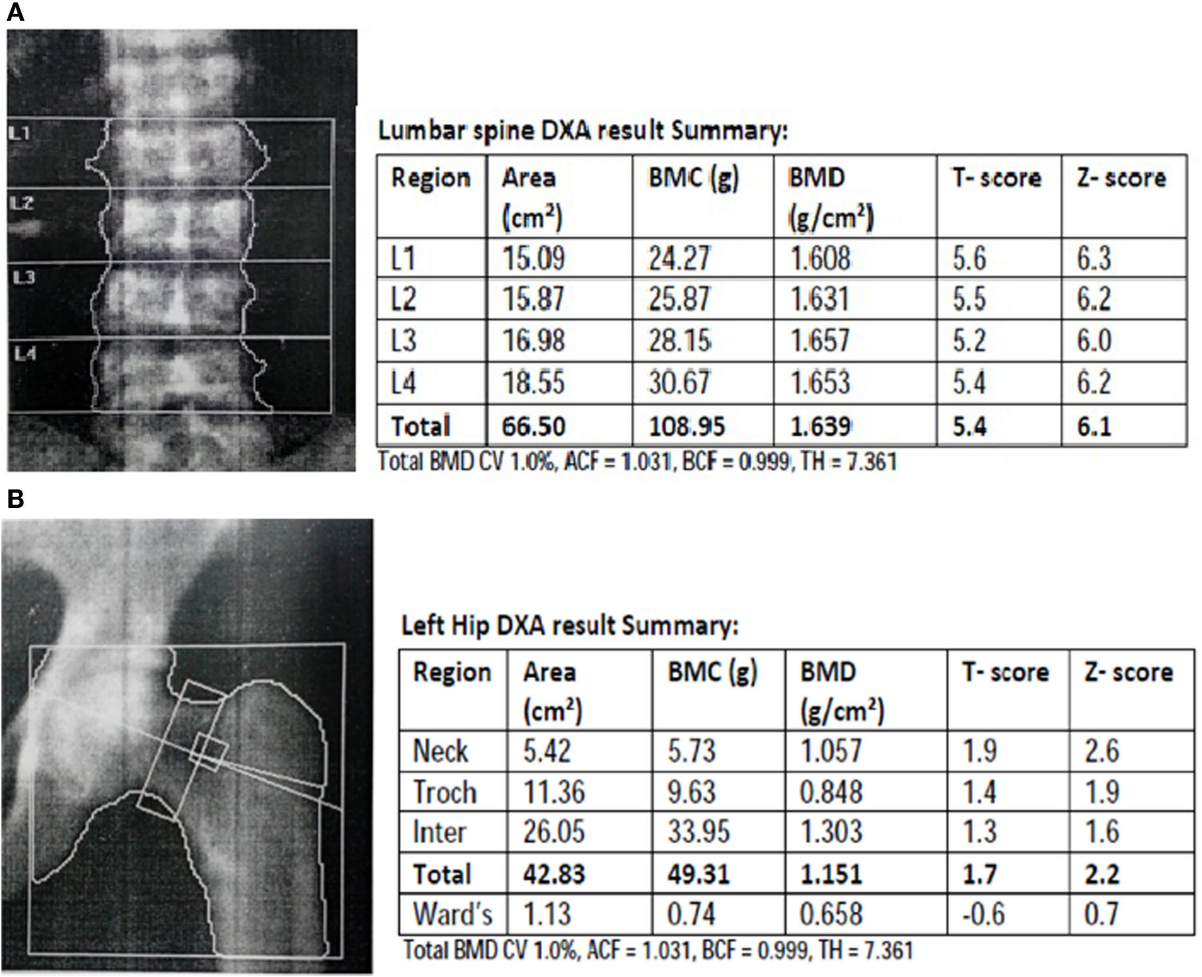
**DXA (bone densitometry) and result summary, (A) spine, (B) hip both showing many fold increase in bone density**.

Suspicion about the systemic fluorosis started during the patient workup when the patient reported that the other family members also suffered from the same kind of complications. After evaluation of family members, we found that they were having clear sign of dental fluorosis in children and skeletal fluorosis in adults and were symptomatic for fluorosis, which includes progressive pain and stiffness of neck, spine and limb joints, muscular weakness, lethargy, and gastrointestinal problems. After radiological and laboratory investigations, family members of the patients were diagnosed and confirmed for fluorosis. As per the family history, it was suspected that the radiological findings of multiple myeloma in the patients may be an unusual manifestation of chronic fluoride toxicity. To confirm the hypothesis, patient’s urinary and serum fluoride concentration were measured by using selective fluoride ion electrode (Orion, Thermo Scientific), and it was found that fluoride level in urine and serum were significantly increased. To further confirm the chronic fluoride toxicity, forearm interosseous membrane calcification was evaluated by X-ray and was positive for the same (Figure 2A), and X-ray hip was also suggestive of increased bone density (Figure 2B). DXA scan (bone densitometry) showed that the bone density increased manyfold, which is also suggestive of skeletal fluorosis (Figures 3A,B).

The MRI findings were suggestive of multiple myeloma but other findings were indicating toward unusual outcome of chronic fluoride toxicity. The gold standard molecular marker for multiple myeloma is the presence of high molecular weight M-protein band in serum and urine, which clearly appears in gel electrophoresis. Therefore, to confirm and/or rule out the multiple myeloma, urinary and serum M-protein electrophoresis was done. However, M-band was absent in urine and serum of the patient, thereby not confirming the diagnosis of multiple myeloma in contrast to MRI findings of multiple myeloma. Multiple myeloma was also ruled out by histopathological examinations. As per the findings such as high concentration of fluoride in urine, serum, and drinking water (Table 1), forearm interosseous membrane calcification, and increased bone density, it was established that this is an unusual manifestation of chronic fluoride toxicity.

**Table 1:** **Fluoride levels in the serum, urine, and drinking water of the patient**.

Fluoride level in	Observed values (ppm)	Normal value (WHO)	Remarks
Urine	9.6	Up to 0.10 ppm	↑
Serum	0.31	Up to 0.02 ppm	↑
Drinking water	10.01	Up to 1.00 ppm	↑

## Discussion

Fluorosis in humans predominantly has dental and skeletal manifestation. In the early stage of chronic fluoride toxicity, patients are asymptomatic or gastrointestinal symptoms may be present. In the advanced stages, skeletal fluorosis causes crippling deformities and neurological complications. Neurological complications occur in 5–10% of cases of skeletal fluorosis (6). These features usually develop after exposure to a high fluoride content for longer period of time (7). Spinal cord involvement occurs in the cervical region followed by thoracic and lumbar region (8). These complications appear mainly due to the compression of the spinal cord and nerve roots by the protruding osteophytes, thickening of the posterior longitudinal ligament, and thickening of the ligamentum flavum resulting in compressive myeloradiculopathy and compressive myelopathy (9, 10), but multiple myeloma-like findings are very rare events and from the best of our knowledge, we are reporting this finding the first time. In fluorosis, the compressive myelopathy has been observed to occur at different levels, but MRI findings suggesting multiple myeloma-like changes was a very unusual finding in fluorosis patient whom we have observed. Though the fluorosis was confirmed by the lab test and by other investigations such as high level of fluoride in body fluid, forearm interosseous membrane ossification/calcification, and increased bone density. M-band in serum and urine was negative, thereby ruling out MRI findings of multiple myeloma and suggesting that fluoride toxicity could probably be bringing out pseudo-multiple myeloma-like changes in the lumbosacral spine. These abovementioned MRI findings with systemic fluorosis are very rare events. Bone marrow aspiration and spinal marrow biopsy are invasive technique and cannot be advised routinely to rule out these types of pseudo-multiple myeloma-like MRI findings in spinal fluorosis. In such type of fluorosis patients if MRI spine is suggestive of multiple myeloma, the spinal biopsy will rule out multiple myeloma but will not suggest fluorosis and yet it is difficult to diagnose. Therefore, this case report will provide a dimension to think about rare spinal fluorosis that may show unusual multiple myeloma-like findings in spinal MRI.

## Conclusion

The multiple myeloma-like MRI findings suggestive of neoplastic bone marrow infiltration and proliferative changes in the spine of fluorosis patients are very unusual, and outmost care should be taken in the diagnosis of such kind of fluoride toxicity. This case report contributes to the literature the possibility of finding multiple myeloma-like changes in the spine due to chronic fluoride toxicity. However, this observation needs to be further investigated in other fluorosis patients with spinal involvement to establish the fact that fluoride toxicity can bring about multiple myeloma-like changes in the spine.

## Author Contributions

Dr. JQ: patient workup and diagnosis. Dr. MA: interpretation of radiological finding and correlation with clinical findings. Ms. SS: fluoride estimation and diagnosis. Dr. AG: patient workup and idea generation about may be the case of fluorosis. Dr. AS: manuscript preparation. Dr. TD: cracked the case and confirmed the diagnosis (Dr. TD died due to his chronic illness).

## Conflict of Interest Statement

The authors declare that the research was conducted in the absence of any commercial or financial relationships that could be construed as a potential conflict of interest.
